# Supplementary material for: Effective use of a horizontally-transferred pathway for dichloromethane catabolism requires post–transfer refinement
Source: eLife. 2014 Nov 24;3:e04279. doi: 10.7554/eLife.04279 (PMC4271186; doi:10.7554/eLife.04279)
Supplement: Figure 3—source data 1. — DOI: http://dx.doi.org/10.7554/eLife.04279.006 [file elife04279s001.docx]

| **Isolate** | **Comments** | **Mutation**  **target** | **Nucleotide**  **mutation** | **Amino acid**  **mutation** | **First**  **identified** |
| --- | --- | --- | --- | --- | --- |
| A1 | Mutation *secY*^A1^ | META1p2172 (*secY*) | Δ(580-591) | Δ(194-197) | Generation 120 |
|  |  | pJM10 (*dcmA*) | c(-122)a |  | Generation 36 |
| A2 | Mutation *secY*^A2^ | META1p2172 (*secY*) | t1223g | I408S | Generation 60 |
| A3 | Mutation *secY*^A3^ | META1p2172 (*secY*) | t572g | I191S | Generation 60 |
| E1 | Resequenced genome, mutation *clcA*^E1^ | Mext_3157 (*clcA*) | Δ(-66→-55) |  | Generation 12 |
| E2 | Resequenced genome, mutations *clcA*^E2^, *besA*^E2^ | Mext_3157 (*clcA*) | c(-50)t |  | Generation 60 |
|  |  | Mext_0368 (*besA*) | a730t | I244F | Generation 120 |
|  |  | Mext_0399 (*hrcA*) | a286g | M96V | N.D. |
| E3 | Resequenced genome, mutation *edgA*^E3^ | Mext_2999 (*edgA*) | g217a | G73S | Generation 36 |
|  |  | Mext_2160 (EF-Tu) | Δ755-777 | ΔT253 | N.D. |
|  |  | Mext_R0061 – Mext_R0062 | Δ14646 bp |  | N.D. |
| P1 | Resequenced genome | Mpop_2144 (*secY*) | t1220g | L407R | N.D. |
|  |  | Mpop_1663 | t488a | I163N | N.D. |
| P2 |  | Mpop_3353 (*clcA*) | a(-99)t |  | N.D. |
| P3 |  | Mpop_3353 (*clcA*) | a(-99)t |  | N.D. |
| N1 | Resequenced genome | Mnod_1928 (*secY*) | t1226a | I409N | Generation 48 |
|  |  | NC_011887 | Δ458,070 bp |  | N.D |
| N2 | Resequenced genome | Mnod_1928 (*secY*) | t575a | I192N | Generation 48 |
| N3 |  | Mnod_1928 (*secY*) | t1226g | I409S | Generation 72 |
| N4 | Resequenced genome | Mnod_1890 (*edgA*) | g208a | G70S | N.D. |
|  |  | Mnod_1441 | g3253a | G1085R | N.D. |

Source data, Figure 3A: Mutations identified during experimental evolution. N.D.: not determined.
