# Supplementary material for: Effective use of a horizontally-transferred pathway for dichloromethane catabolism requires post–transfer refinement
Source: eLife. 2014 Nov 24;3:e04279. doi: 10.7554/eLife.04279 (PMC4271186; doi:10.7554/eLife.04279)
Supplement: Figure 6—source data 1. — Closest relative was determined based on the nearest BLAST hit to the 16S rRNA sequence of the isolate. DOI: http://dx.doi.org/10.7554/eLife.04279.016 [file elife04279s002.docx]

| **Strain identifier** | **Closest relative** | **Fitness with pJM10 (*dcmA*)** | **Fitness with pJM83 (*dcmA*/*clcA*)** |
| --- | --- | --- | --- |
| *M. extorquens* AM1 |  | -0.06 ± 0.02 | 0.37 ± 0.01 |
| *M. extorquens* PA1 |  | 0.20 ± 0.02 | 0.71 ± 0.03 |
| *M. extorquens* CM4 |  | 0.14 ± 0.03 | 0.68 ± 0.05 |
| *M. extorquens* BJ001 |  | 0.37 ± 0.04 | 0.72 ± 0.02 |
| *M. nodulans* ORS 2060 |  | 0.43 ± 0.01 | 0.71 ± 0.03 |
| *M. radiotolerans* JCM 2831 |  | 0.37 ± 0.01 | 0.51 ± 0.01 |
| L1 | *Methylobacterium sp. A4* | 0.06 ± 0.05 | 0.49 ± 0.01 |
| D21 | *M. fujisawaense* | 0.12 ± 0.01 | 0.36 ± 0.03 |
| D23 | *M. extorquens* | 0.13 ± 0.01 | 0.23 ± 0.02 |
| D24 | *M. extorquens* | -0.02 ± 0.01 | 0.28 ± 0.04 |
| J-4-1 | *M. bullatum* | -0.08 ± 0.02 | 0.64 ± 0.01 |
| C-7-2 | *M. oxalidis* | 0.00 ± 0.07 | 0.41 ± 0.03 |
| C-7-1 | *M. extorquens* | 0.21 ± 0.02 | 0.63 ± 0.01 |
| C-2-3 | *M. extorquens* | 0.01 ± 0.03 | 0.25 ± 0.02 |
| G-1-1 | *M. braciatum* | 0.34 ± 0.01 | 0.37 ± 0.01 |
| M-1-1 | *M. trifolii* | 0.27 ± 0.05 | 0.48 ± 0.03 |

Source data, Figure 6: Environmental *Methylobacterium* strains characterized with the dual expression plasmid. Closest relative was determined based on the nearest BLAST hit to the 16S rRNA sequence of the isolate.
