# Supplementary material for: Effective use of a horizontally-transferred pathway for dichloromethane catabolism requires post–transfer refinement
Source: eLife. 2014 Nov 24;3:e04279. doi: 10.7554/eLife.04279 (PMC4271186; doi:10.7554/eLife.04279)
Supplement: Supplementary file 1. — DOI: http://dx.doi.org/10.7554/eLife.04279.017 [file elife04279s003.docx]

| **Designation** | **Strain ID** | **Genotype** | **Reference** |
| --- | --- | --- | --- |
| AM1 | CM3120 | *M. extorquens* AM1 Δ*cel* *katA*::*mCherry* | Michener et al., 2014 |
| PA1 | CM3839 | *M. extorquens* PA1 Δ*cel* *hpt*::*mCherry* | Michener et al., 2014 |
| DM4 Δ*dcmA* | CM4250 | *M. extorquens* DM4 Δ*dcmA* *hpt*::*Venus* | Michener et al., 2014 |
| AM1-C *secY*^A1^ | CM4251 | *M. extorquens* AM1 Δ*cel* *katA*::*mCherry secYΔ194-197* | This work |
| AM1-V *secY*^A2^ | CM4252 | *M. extorquens* AM1 Δ*cel* *katA*::*Venus secY(I191S)* | This work |
| AM1-C *secY*^A3^ | CM4253 | *M. extorquens* AM1 Δ*cel* *katA*::*mCherry secY(I408S)* | This work |
| AM1-C *clcA*^E1^ | CM4383 | *M. extorquens* AM1 Δ*cel* *katA*::*mCherry clcA*Δ*(-66→-55)* | This work |
| AM1-C *clcA*^E2^ | CM4384 | *M. extorquens* AM1 Δ*cel* *katA*::*mCherry clcA(C(-50)T)* | This work |
| PA1-C *secY*^A1^ | CM4254 | *M. extorquens* PA1 Δ*cel* *hpt*::*mCherry secYΔ194-197* | This work |
| PA1-C *clcA*^E1^ | CM4385 | *M. extorquens* PA1 Δ*cel* *hpt*::*mCherry clcA*Δ*(-66→-55)* | This work |
| PA1-C *clcA*^E2^ | CM4386 | *M. extorquens* PA1 Δ*cel* *hpt*::*mCherry clcA(C(-50)T)* | This work |
| PA1-C *besA*^E2^ | CM4387 | *M. extorquens* PA1 Δ*cel* *hpt*::*mCherry besA(I244F)* | This work |
| PA1-C *secY*^A1^*clcA*^E1^ | CM4388 | *M. extorquens* PA1 Δ*cel* *hpt*::*mCherry secYΔ194-197 clcA*Δ*(-55→-66)* | This work |
| PA1-C *besA*^E2^*clcA*^E2^ | CM4389 | *M. extorquens* PA1 Δ*cel* *hpt*::*mCherry clcA(C(-50)T) besA(I244F)* | This work |
| PA1-C *clcA*^D0^ | CM4390 | *M. extorquens* PA1 Δ*cel* *hpt*::*mCherry clcA(140 bp from DM4)* | This work |
| DM4 *clcA*^E0^ | CM4391 | *M. extorquens* DM4 Δ*dcmA clcA(140 bp from PA1)* | This work |
| PA1 *clcA*^E1^ | CM4392 | *M. extorquens* PA1 Δ*cel* *clcA*Δ*(-66→-55)* | This work |
| PA1-C *edgA*^E3^ | CM4393 | *M. extorquens* PA1 Δ*cel* *hpt*::*mCherry edgA(G73S)* | This work |
| PA1-C *edgA*^E3^ *clcA*^E1^ | CM4394 | *M. extorquens* PA1 Δ*cel* *hpt*::*mCherry edgA(G73S) clcA*Δ*(-66→-55)* | This work |

Supplementary File 1A: Strains used in this study.

| **Plasmid** | **Description** | **Reference** |
| --- | --- | --- |
| pJM10 | *dcmA kanR2* | Michener et al., 2014 |
| pJM40 | *P_tac_*-*mCherry*-*pHluorin* *dcmA tetA* | Michener et al., 2014 |
| pPS04 | *oriT^RP4^ sacB kanR2* | Michener et al., 2014 |
| pJM83 | *dcmA* *P_tac­_-clcA tetA* | This work |
| pJM61 | pPS04 AM1 *secY*^A3^ | This work |
| pJM62 | pPS04 AM1 *secY*^A1^ | This work |
| pJM64 | pPS04 AM1 *secY*^A2^ | This work |
| pJM66 | pPS04 PA1 *secY*^A1^ | This work |
| pJM67 | pPS04 PA1 *secY*^A3^ | This work |
| pJM72 | pPS04 PA1 *clcA*^E1^ | This work |
| pJM73 | pPS04 PA1 *clcA*^E2^ | This work |
| pJM74 | pPS04 PA1 *besA*^E2^ | This work |
| pJM75 | pPS04 AM1 *clcA*^E1^ | This work |
| pJM76 | pPS04 AM1 *clcA*^E2^ | This work |
| pJM88 | pPS04 PA1 *clcA*^D0^ | This work |
| pJM89 | pPS04 DM4 *clcA*^E0^ | This work |
| pJM98 | pPS04 PA1 *edgA*^E3^ | This work |

Supplementary File 1B: Plasmids used in this study.
